# The Changing Role of Chemotherapy in Locoregionally Advanced Nasopharyngeal Carcinoma: A Updated Systemic Review and Network Meta-Analysis

**DOI:** 10.3389/fonc.2018.00597

**Published:** 2018-12-19

**Authors:** Mei Liu, Wei You, Yi-Bing Song, Ji-Dong Miao, Xiu-Bo Zhong, Dian-Kun Cai, Lun Xu, Lu-Feng Xie, Yang Gao

**Affiliations:** Department of Oncology, Zigong NO. 4 People's Hospital, Zigong, China

**Keywords:** nasopharyngeal carcinoma, concurrent chemoradiotherapy, induction chemotherapy, adjuvant chemotherapy, network meta-analysis

## Abstract

**Background and Objective:** Both induction chemotherapy (IC) followed by concurrent chemoradiotherapy (CCRT; IC+CCRT) and CCRT plus adjuvant chemotherapy (AC; CCRT+AC) are standard treatments for advanced nasopharyngeal carcinoma (NPC). However, no prospective randomized trials comparing these two approaches have been published yet. We conducted this network meta-analysis to address this clinical question.

**Method:** We recruited randomized clinical trials involving patients with advanced NPC randomly allocated to IC+CCRT, CCRT+AC, CCRT, or radiotherapy (RT) alone. Pairwise meta-analysis was first conducted, then network meta-analysis was performed using the frequentist approach. Effect size was expressed as hazard ratio (HR) and 95% confidence interval (CI).

**Results:** Overall, 12 trials involving 3,248 patients were recruited for this study, with 555 receiving IC+CCRT, 840 receiving CCRT+AC, 1,039 receiving CCRT, and 814 receiving radiotherapy (RT) alone. IC+CCRT achieved significantly better overall survival ([HR], 0.69; 95% [CI], 0.51–0.92), distant metastasis-free survival (HR, 0.58; 95% CI, 0.44–0.78), and locoregional recurrence-free survival (HR, 0.67; 95% CI, 0.47–0.98) than CCRT. However, survival outcomes did not significantly differ between IC+CCRT and CCRT+AC, or between CCRT+AC and CCRT arms for all the endpoints. As expected, RT alone is the poorest treatment. In terms of P-score, IC+CCRT ranked best for overall survival (96.1%), distant metastasis-free survival (99.0%) and locoregional recurrence-free survival (87.1%).

**Conclusions:** IC+CCRT may be a better and more promising treatment strategy for advanced NPC; however, head-to-head randomized trials comparing IC-CCRT with CCRT-AC are warranted.

## Background

Nasopharyngeal carcinoma (NPC) arises from the nasopharynx epithelium and achieves the highest incidence among all head and neck cancers in China (1). Worldwide, NPC exhibits an extremely unbalanced distribution with an incidence of 20–50 per 100,000 in Southern China but <1 per 100,000 in most western countries (2, 3). As constrained by its complicated anatomical location, surgery is not available and radiotherapy (RT) has become the only radical curative treatment for NPC. As NPC is also highly sensitive to chemotherapeutic agents, incorporation of chemotherapy with RT has been established as the standard care for stage II-IVA disease. Notably, patients with early disease usually achieve excellent survival outcomes while prognosis of advanced disease still remains poor (4).

Upon the publishing of Intergroup 0099 trial in 1998, this milestone study has established concurrent chemoradiotherapy (CCRT) plus adjuvant chemotherapy (AC) as the standard regimen for advanced NPC since it could provide a 31% increase in overall survival (OS) (5). However, many subsequent studies demonstrated that AC additional to CCRT may be useless (6–8). More importantly, AC brought severe toxicities and many patients could not complete the assigned cycles, which constrains its wide clinical application. Given this, other intensive treatment strategies should be developed. Recently, there is increasing amount of evidence showing that induction chemotherapy (IC), delivered before radiotherapy, is also an effective and promising treatment strategy as it has better compliance rates and facilitates early eradication of micrometastases (9–12). Based on these findings, the National Comprehensive Cancer Network (NCCN) guidelines recommend IC plus CCRT as one of the standard treatments for stage II-IVA disease. However, it still remains unclear which chemotherapy sequence is better as we lack head-to-head trials comparing IC+CCRT with CCRT+AC. In view of the urgent need for effective and less toxic therapies, we conducted this network meta-analysis to compare IC+CCRT with CCRT+AC through extracting data from published clinical randomized trials.

## Results

### Baseline Information of Recruited Trials

**Figure 1:**
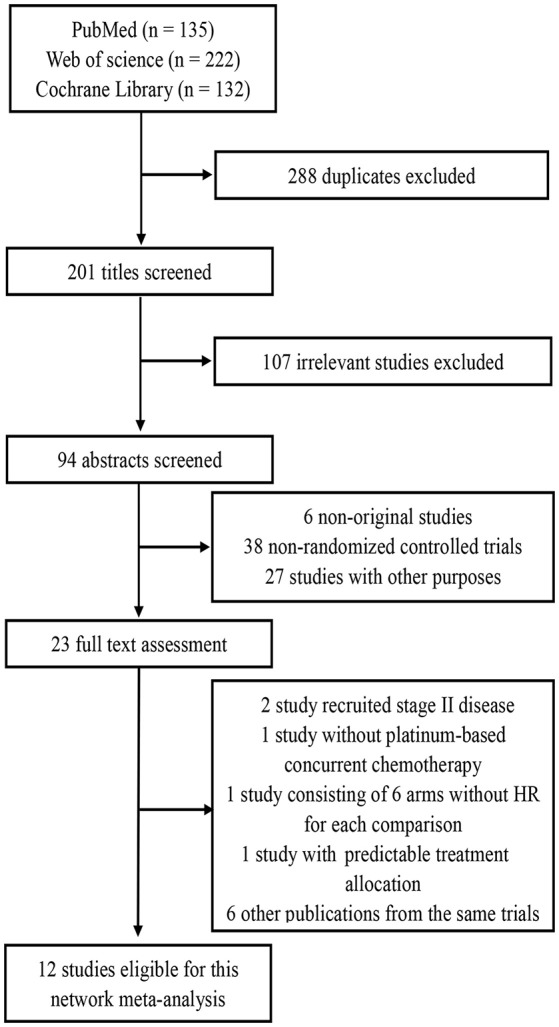
Flow chart of study inclusion.

By the last literature searching (May 2018), we in total identified 24 potentially eligible clinical trials. Flow chart of studies inclusion was presented in Figure 1. The study by Lin et al. (13) was not included because HRs and 95% CI was not provided in original text. Two studies involving stage II NPC were excluded (14, 15). Due to the one-side 95% CI reported in the study by Tan et al. (16) and unknown HR for each treatment comparison in the study by Lee et al. (17), we therefore excluded these two studies. We also excluded the study by Kwong et al. (18) because uracil + tegafur was used as the concurrent chemotherapy regimen; however, this study would be included in the sensitivity analysis. Additionally, six studies updated their long follow-up data: Chan et al. (19, 20), Lee et al. (21, 22), Lee et al. (23, 24), Chen et al. (25, 26), Chen et al. (7, 27), and Zhang et al. (28, 29). Finally, 12 studies (5, 9–12, 19, 22, 23, 26–28, 30) were included for the current study. Notably, we excluded two treatment arms receiving accelerated-fraction radiotherapy in the study by Lee et al. (23, 24) because they did not meet the inclusion criterion of conventional-fraction radiotherapy. The basic information of the 12 studies are summarized in Table 1. In total, 3,248 patients were randomly allocated with 555 receiving IC+CCRT, 840 receiving CCRT+AC, 1,039 receiving CCRT, and 814 receiving RT alone. Quality assessment of the 12 studies was summarized in Supplementary Table S1.

**Table 1:** Summary of basic information of the 12 studies included in this network meta-analysis.

**Study**	**No. of patients**	**Study time**	**Median follow-up duration (months)**	**Patient stage**	**Radiotherapy**	**Chemotherapy**		
						**Induction**	**Concurrent**	**Adjuvant**
**IC + CCRT vs. CCRT**								
Hui et al. (10)	65	2002–2004	51.6	AJCC III-IVB, T1-4, N0-3	66 Gy/33f at 2 Gy/f/day (5f/qw) + additional boost of 20 Gy/10f to parapharyngeal	Docetaxel 75 mg/m^2^ d1 + DDP 75 mg/m^2^ d1 q3w × 2	40 mg/m^2^ d1 qw × 8	None
Frikha et al. (12)	83	2009–2012	43.1	AJCC T2b-4, N1-3	70 Gy/35f at 2 Gy/f/day (5f/qw)	Docetaxel 75 mg/m^2^ d1 + Cisplatin 75 mg/m^2^ d1+ 5-FU 750 mg/m^2^ d1-5 q3w × 3	DDP 40 mg/m^2^ weekly for 8 weeks	None
Sun et al. (11)	480	2011–2013	45	AJCC III-IVB, except T3-4N0	≥ 66 Gy at 2.00–2.35 Gy/f/day for 6–7 weeks	Docetaxel 60 mg/m^2^ d1 + DDP 60 mg/m^2^ d1 + Fu 600 mg/m^2^/day d1-5 civ q3w × 3	100 mg/m^2^ d1 q3w × 3	None
Cao et al. (9)	476	2008–2015	50	AJCC III-IVB, except T3N0-1	≥ 66 Gy at 2.0-2–33 Gy/f/day	DDP 80 mg/m^2^ d1 + Fu 800 mg/m^2^/day d1-5 civ q3w × 3	80 mg/m^2^ d1 q3w × 3	None
**CCRT + AC vs. RT**								
Al-Sarraf et al. (5)	193^a^	1989–1995	32.4	AJCC III-IV	66–70 Gy at 1.8–2.0 Gy/f/day (5f/qw)	None	DDP 100 mg/m^2^ d1 q3w × 3	DDP 80 mg/m^2^ d1 + Fu 1000 mg/m^2^/day d1-4 civ q3w × 4
Wee et al. (30)	221	1997–2003	38.4	AJCC III-IV, T3-4Nx or TxN2-3	70 Gy/35f at 2 Gy/f/day (5f/qw) for 7 weeks	None	DDP 25 mg/m^2^/day for 4 days or 30/30/40 mg/m^2^/day for 3 days q3w × 3	DDP 20 mg/m^2^/day for 4 days + Fu 1000 mg/m^2^/day d1-4 q3w × 3
Lee et al. (21, 22)	348	1999–2004	70.8	AJCC III-IV, any T, N2-3	≥ 66 Gy at 2.0 Gy/f/day (5f/qw) + additional boosts to parapharyngeal space, primary, or nodal sites when indicated not exceeding 20 Gy	None	100 mg/m^2^ d1 q3w × 3	DDP 80 mg/m^2^ d1 + 1000 mg/m^2^/day d1-d4 civ q4w × 3
Lee et al. (23, 24)	93	1999–2004	75.6	AJCC III-IV, T3-4N0-1	≥ 66 Gy at 2.0 Gy/f/day (5f/qw) + additional boosts to parapharyngeal space, primary, or nodal sites when indicated not exceeding 20 Gy	None	100 mg/m^2^ d1 q3w × 3	DDP 80 mg/m^2^ d1 + 1000 mg/m^2^/day d1-d4 civ q4w × 3
Chen et al. (25, 26)	316	2002–2005	70	AJCC III-IV, T1-4, N0-3	≥ 68 Gy at 2.0 Gy/f/day (5f/qw) for 7 weeks + additional boost in case of parapharyngeal extension, residual neck and/or nasopharyngeal tumor	None	100 mg/m^2^ d1 q3w × 3	DDP 80 mg/m^2^ d1 + Fu 800 mg/m^2^/day d1-5 civ q3w × 3
**CCRT+AC vs. CCRT**								
Chen et al. (7, 27)	508	2006–2010	68.4	AJCC III-IVB except T3-4N0	≥ 66 Gy at 2.0-2.27 Gy/f/day (5f/qw) for 6–7 weeks	None	DDP 40 mg/m^2^ d1 weekly for up to 7 weeks	DDP 80 mg/m^2^ d1 + Fu 800 mg/m^2^/day d1-5 civ q4w × 3
**CCRT vs. RT**								
Chan et al. (19, 20)	350	1994–1997	66	Ho's N2-3 or N1 with nodal size ≥ 4cm	66 Gy + additional boost in case of parapharyngeal extension, residual neck, or nasopharyngeal tumor	None	DDP 40 mg/m^2^ d1 weekly for 8 weeks	None
Zhang et al. (28, 29)	115	2001–2003	114	AJCC III-IV, any T, N2-3	70-74 Gy at 2 Gy/f/day (5f/qw) + additional boost in case of parapharyngeal extension, residual neck, or nasopharyngeal tumor	None	Oxaliplatin 70 mg/m^2^ d1 weekly for 6 weeks	None

*CCRT, concurrent chemoradiotherapy; IC, induction chemotherapy; AC, adjuvant chemotherapy; RT, radiotherapy; AJCC, American Joint Committee on cancer; f, fraction; DDP, cisplatin; Fu, fluorouracil; civ, continuous i.v.; q3w, every 3 weeks; q4w, every 4 weeks; AUC, area under concentration-time curve. 2D-RT, two-dimensional radiotherapy; IMRT, intensity-modulated radiotherapy*.

a *193 patients were registered, but only 147 patients were analyzed*.

### Traditional Pairwise Comparison

**Figure 2:**
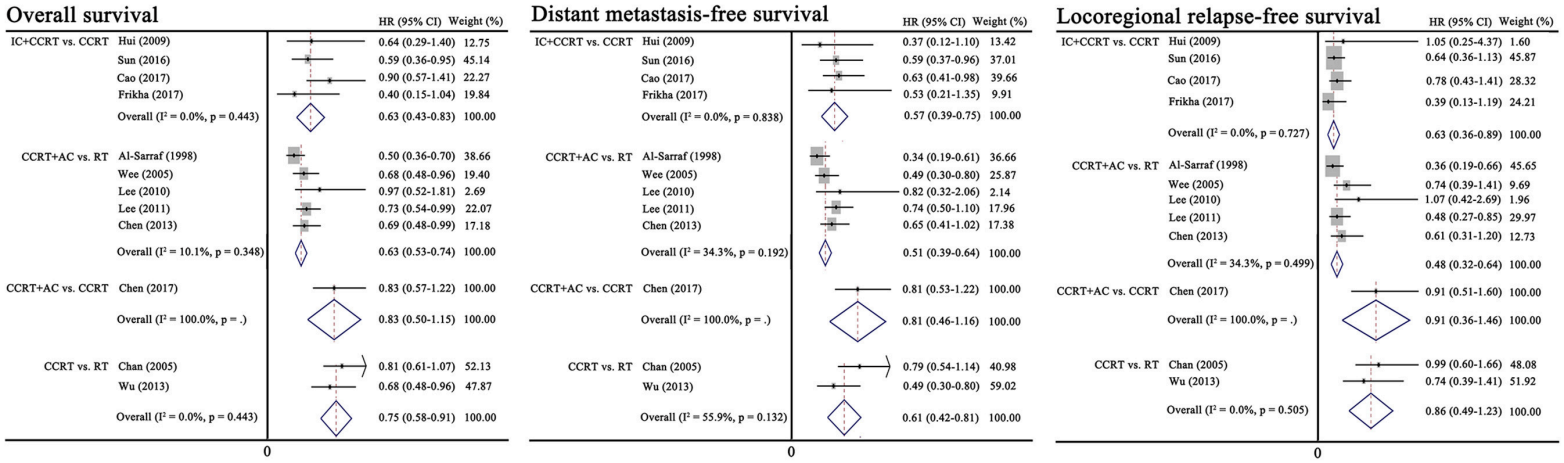
Results of traditional pairwise meta-analysis. CCRT, concurrent chemoradiotherapy; IC, induction chemotherapy; AC, adjuvant chemotherapy; RT, radiotherapy; HR, hazard ratio; CI, confidence interval.

Figure 2 presents the results of pairwise meta-analysis. Heterogeneity between treatment arms only existed in CCRT vs. RT for DMFS (*I*^2^ = 55.9%), and a random-effects model was then applied. Compared with CCRT, IC+CCRT was associated with significantly improved OS (HR, 0.65; 95% CI, 0.43–0.83), DMFS (HR, 0.57; 95% CI, 0.39–0.75), and LRFS (HR, 0.63; 95% CI, 0.36–0.89). Undoubtedly, CCRT+AC achieved better OS (HR, 0.63; 95% CI, 0.53–0.74), DMFS (HR, 0.51; 95% CI, 0.39–0.64), and LRFS (HR, 0.48; 95% CI, 0.32–0.64) than RT alone. Similarly, CCRT could prolong OS (HR, 0.75; 95% CI, 0.58–0.91) and DMFS (HR, 0.61; 95% CI, 0.42–0.81) compared with RT alone. Consistent with the original study, no significant differences between CCRT+AC and CCRT were observed in terms of OS, DMFS and LRFS.

### Multiple Network Comparison

**Figure 3:**
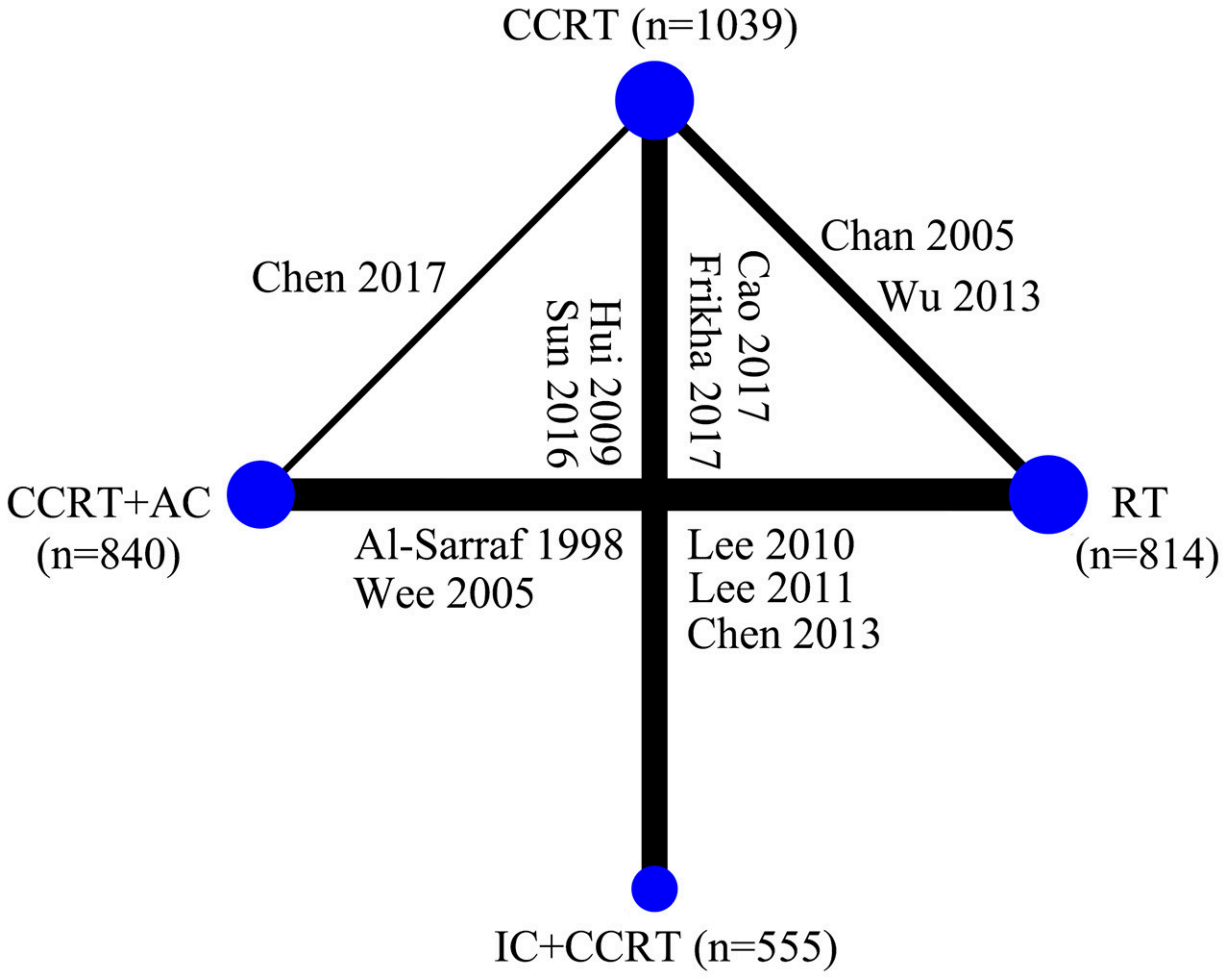
Graphical presentation of the trial network for overall survival. The width of the lines between nodes is proportional to the number of comparisons. Only two treatment arms receiving conventional-fraction radiotherapy in the study by Lee et al. (23) were included in this study. CCRT, concurrent chemoradiotherapy; IC, induction chemotherapy; AC, adjuvant chemotherapy; RT, radiotherapy.

**Figure 4:**
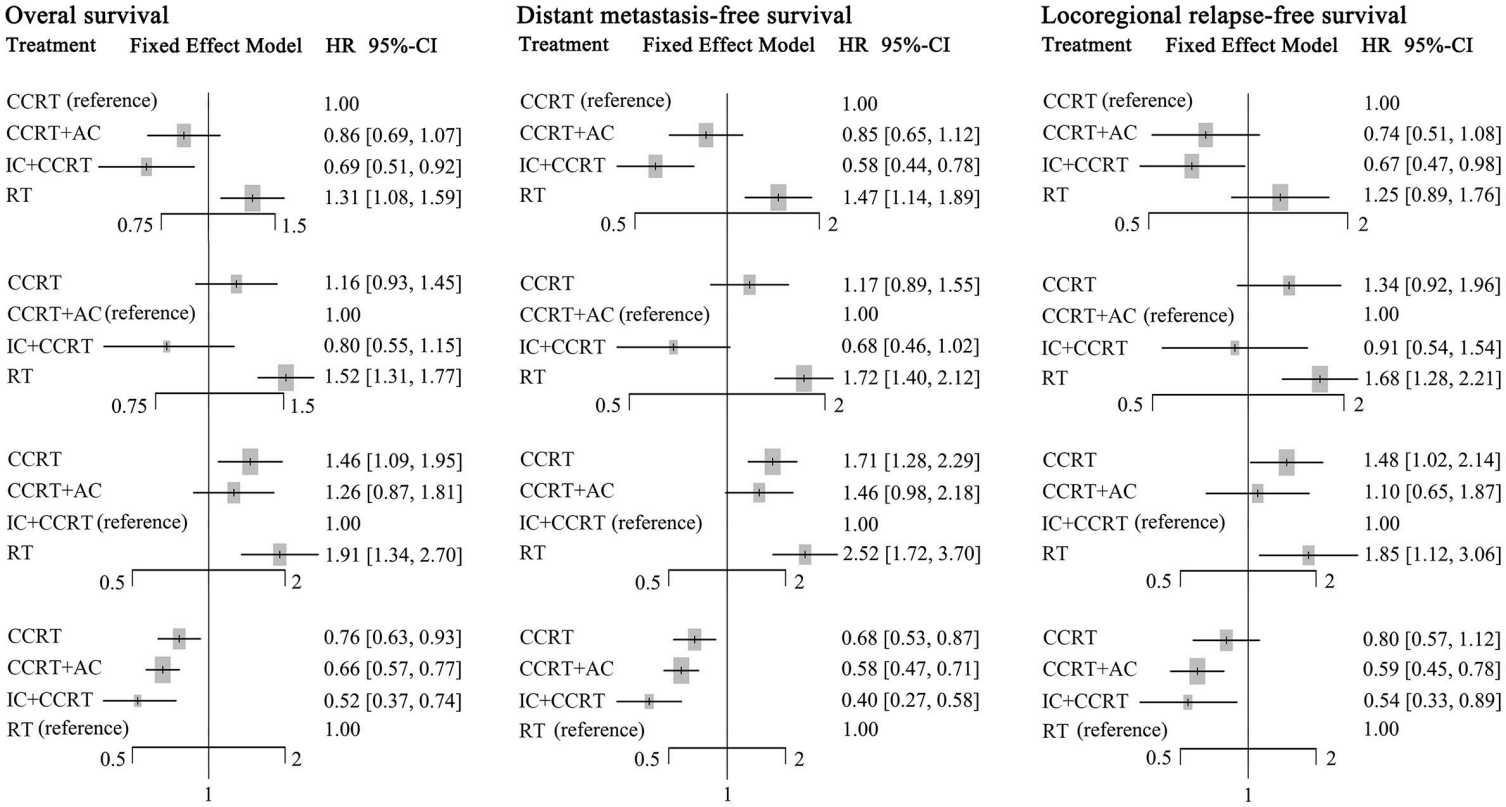
Forest plot of network meta-analysis for overall survival, distant metastasis-free survival, and locoregional recurrence-free survival with different reference groups. CCRT, concurrent chemoradiotherapy; IC, induction chemotherapy; AC, adjuvant chemotherapy; RT, radiotherapy.

Figure 3 presented the network analysis of the four treatment arms (IC+CCRT, CCRT+AC, CCRT, and RT). In the multiple comparison, CCRT arm was treated as the reference group, and results network meta-analysis are summarized in Table 2. There is no inconsistency or heterogeneity neither between nor within studies (*P* > 0.1 for all rates). Thus, a fixed-effects model was used. The forest plots of multiple treatment comparisons with different reference groups were presented in Figure 4.

**Table 2:** Results of multiple treatment comparison for the three endpoints.

**Treatment arm**	**OS**	**DMFS**	**LRFS**
*P*-value of Overall heterogeneity/inconsistency	0.51	0.44	0.55
*P*-value of heterogeneity (within designs)	0.41	0.35	0.55
*P*-value of heterogeneity (between designs)	0.82	0.74	0.34
**CCRT**			
HR	1.00	1.00	1.00
P-score (%)	36.5	37.6	32.7
**CCRT+AC**			
HR (95% CI)	0.86 (0.69–1.07)	0.85 (0.65–1.12)	0.74 (0.51–1.08)
P-score (%)	67.3	63.4	76.6
**IC+CCRT**			
HR (95% CI)	0.69 (0.51–0.92)	0.58 (0.44–0.78)	0.67 (0.47–0.98)
P-score (%)	96.1	99.0	87.1
**RT**			
HR (95% CI)	1.31 (1.08–1.59)	1.47 (1.14–1.89)	1.25 (0.89–1.76)
*P*-score (%)	0.1	0.4	3.6

*OS, overall survival; DMFS, distant metastasis-free survival; LRFS, locoregional recurrence-free survival; CCRT, concurrent chemoradiotherapy; AC, adjuvant chemotherapy; IC, induction chemotherapy; RT, radiotherapy. HR, hazard ratio; CI, confidence interval*.

*Fixed-effects model was used for overall survival, distant metastasis-free survival and locoregional recurrence-free survival*.

Compared to CCRT, IC+CCRT achieved significantly better OS (HR, 0.69; 95% CI, 0.51–0.92), DMFS (HR, 0.58; 95% CI, 0.44–0.78), and LRFS (HR, 0.67; 95% CI, 0.47–0.98). However, no significant survival differences were found between CCRT+AC and CCRT, or CCRT+AC and IC+CCRT (Supplementary Tables S2–S4). Notably, RT alone always led to significantly poorer survival outcomes compared with the other three treatments except RT vs. CCRT for LRFS (HR, 1.25; 95% CI, 0.89–1.76).

The corresponding P-scores of IC+CCRT, CCRT+AC, CCRT, and RT treatment arms were 96.1%, 67.3, 36.5, and 0.1% for OS; 99.0, 63.4, 37.6, and 0.4% for DMFS; 87.1, 76.6, 32.7, and 3.6% for LRFS, indicating IC+CCRT has the highest probability of being the best treatment in terms of OS, DMFS, and LRFS.

### Sensitivity Analysis

We further performed sensitivity analysis after including the study by Kwong et al. (18) to validate our findings; and the results are shown in the Supplementary Results (Supplementary Figures S1–S3, Supplementary Tables S5,S6). Notably, the conclusions remained valid after including this study. More importantly, IC+CCRT was even found to be superior to CCRT+AC with regard to DMFS (HR, 0.63; 95% CI, 0.43–0.93). Similarly, IC+CCRT still provided the highest benefit on OS, DMFS, and LRFS. These results indicated that IC+CCRT may be better than CCRT+AC.

## Discussion

In our current study, we applied frequentist method to conduct multiple treatment comparisons between IC+CCRT, CCRT+AC, CCRT, and RT in advanced NPC based on all available information extracted from the published studies. We found that IC+CCRT was superior to CCRT and RT, and provided the largest OS, DMFS, and LRFS benefits. While no significant difference was observed between IC+CCRT and CCRT+AC. Further sensitivity analysis after including the study by Kwong et al. (18) also yield similar results. Notably, no inconsistency and heterogeneity were observed between these comparisons for all end-points, indicating that our findings are robust.

The role of chemotherapy in managing advanced NPC has changed greatly over the last two decades. Before the Intergroup 0099 study (5), radiotherapy alone is the only care for both early and advanced disease. Later on, CCRT+AC was proven better than RT alone in improving OS and this regimen has deemed the standard treatment for advanced NPC. However, a meta-analysis conducted by Baujat et al. (6) revealed this survival benefit mainly came from concurrent chemotherapy during RT. Moreover, the study by Chen et al. (7) found that AC additional to CCRT may be useless and this conclusion was further proven by long-term follow-up outcomes (27). Consequently, CCRT with or without AC was recommended by the NCCN guidelines. Although this regimen was applied, distant metastasis still remains the main failure pattern for advanced NPC (31). Therefore, novel treatments like IC was introduced. However, we still know little about the efficacy difference between these treatment modalities. In our study, we aimed at addressing this issue.

IC+CCRT achieved significantly better OS, DMFS, and LRFS than CCRT in both the pairwise and network meta-analyses, Which was different from the findings by Ribassin-Majed et al. (32). Undoubtedly, the inclusion of the lastest three IC studies (9, 11, 12) could add the weight of IC+CCRT in the network loop, resulting in better efficacy than CCRT alone. Another possible reason contributing to the survival difference between these two groups may be the difference of radiotherapy technique since almost all patients in IC+CCRT arm received intensity-modulated radiotherapy (IMRT) while some patients in CCRT arm received conventional radiotherapy. Therefore, we could conclude that IC+CCRT is better than CCRT and should be considered prior to CCRT. Although IC+CCRT was not found to be better than CCRT in other head and neck cancers (33), we should not apply this result to NPC because NPC has extremely different biological behaviors and is more sensitive to radiotherapy and chemotherapy compared with other head and neck cancers. It should be noted that the delivery of IC should be selective. Recently, two studies (34, 35) revealed additional IC to CCRT may be useless in T3-4N0-1 patients, indicating that only high-risk patients (defined as patients with N2-3 category, overall stage IVA or high pre-treatment Epstein-Barr virus DAN load) may benefit from IC. Moreover, the IC regimen also plays a key role. Docetaxel plus cisplatin with fluorouracil (TPF) has been proven to be superior to PF in head and neck cancer (36–38). Moreover, gemcitabine with cisplatin (GP) has been proven superior to PF in recurrent or metastatic NPC (39). Therefore, selection of effective IC regimens for high-risk patients should be a priority.

Similar to the results of original studies (7, 27), survival outcomes did not significantly differ between CCRT+AC and CCRT treatment arms, suggesting the value of adding AC to CCRT may be limited. Notably, all the included studies regarding CCRT+AC used the recommended AC regimen, cisplatin with fluorouracil. However, this combined AC regimen did not improve survival outcomes compared with either single-agent regimen individually in head and neck cancer (40). In addition, compliance to three cycles of AC was poor (5, 7, 21, 25, 30) and many patients also require dose reductions. Therefore, it is reasonable to infer the adjuvant PF regimen additional to CCRT is not good enough to further improve survival outcomes. Other regimens like GP or single-agent maintenance therapy should be further investigated.

CCRT may be inadequate for high-risk patients with advanced NPC; additional cycles of chemotherapy are worth being investigated (41). Therefore, either IC+CCRT or CCRT+AC may be a better choice than CCRT alone. However, we lack head-to-head clinical trials comparing IC+CCRT with CCRT+AC. In this study, survival outcomes did not differ significantly between CCRT+AC and IC+CCRT for any end-point. However, after including the study by Kwong et al. (18), IC+CCRT achieved better DMFS than CCRT-AC, which was inconsistent with the finding by Ribassin-Majed et al (32). The main reason as we discuss above is the inclusion of three new trials which achieved positive results and added the weight of IC+CCRT in the network loop. Therefore, IC+CCRT may be a little better than, or at least as efficacious as, CCRT+AC. In light of efficacy, it is reasonable to recommend IC+CCRT as the preferred treatment for advanced NPC. There may be another concern about IC+CCRT that IC may affect compliance with subsequent radiotherapy. Since our study was not based on individualized patient data, we, therefore, could not conclude on this. However, from historical data (9, 11, 12, 16), IC may have no impact on the compliance to radiotherapy. Actually, patients receiving or not receiving IC have same completion rate of radiotherapy at clinical practice. However, it should be pointed that compliance of concomitant chemotherapy might be impacted by IC.

Undoubtedly, RT was always poorer than IC+CCRT, CCRT+AC, and CCRT for almost all end-points. Thus, RT alone should not be recommended whenever possible. Notably, the rank of each treatment was indicated by the P-score in multiple treatment comparison. Although differences in effect size between different treatment arms were small and non-significant, a treatment ranking probability would still have been generated without definitive statistical meaning. Therefore, we should interpret the P-score discreetly, and clinical treatment strategies should not only refer to it.

Our study also had limitations: HRs and corresponding 95% CIs were mainly extracted from the original studies, which may produce reporting bias. Radiotherapy technique varied between different treatment arms which may affect the results of our study, and this issue should be solved by future individualized study data. Also, the role of hyperfractionated or accelerated hyperfractionated radiotherapy needs further investigation. Moreover, endpoints did not include PFS as the definitions of PFS varied between studies. To minimize these limitations, we set strict inclusion criteria and three investigators independently reviewed and extracted data. Furthermore, sensitivity analysis confirmed the findings were valid.

## Materials and Methods

### Literature Searching Strategy

First, we searched the English datasets including PubMed, Web of Science, and the Cochrane Library using the following items: “nasopharyngeal carcinoma” and “induction chemotherapy” or “neoadjuvant chemotherapy” or “adjuvant chemotherapy” or “concurrent chemoradiotherapy” or “radiotherapy.” Study type was restricted to clinical trial. Two investigators (ML and WY) performed the searching independently to identify all potentially eligible studies. Furthermore, we will also retrieve the National Knowledge Infrastructure and WanFang database to include any related Chinese references. Supplementary Method showed the detailed process of literature searching. The institutional ethical review board of Zigong NO. 4 People's Hospital approved our current study. All study methods were performed in accordance with our center guidelines.

### Study Inclusion Criteria

Brief inclusion criteria of our study were as follow: (1) newly diagnosed advanced NPC without metastasis; (2) randomized controlled phase II/III trials; (3) patients received conventional-fractionation and radical radiotherapy; (4) concurrent chemotherapy should be platinum-based regimens. Supplementary Method presented the detailed information on study inclusion criteria. In our present study, we mainly recruited four treatment arms (CCRT+AC, IC+CCRT, CCRT, and RT alone) to conduct multiple network comparisons.

### Study Review and Data Acquisition

In order to assess the quality of the recruited trials, the following items were reviewed to score each study according to Jadad/Oxford quality scoring system(42): randomization procedure, blinding principle, intention-to-treat principle, allocation concealment, and patient dropout. The study information such as included patients, study time, radiotherapy, and chemotherapy regimens, follow-up duration, and survival outcomes were extracted. Three investigators (ML, WY, and J-DM) performed the review process and data acquisition separately, and any discrepancies would be resolved by consensus.

### Study Endpoints

Survival outcomes were shown as hazard ratios (HRs) and corresponding confidence intervals (CIs) which were extracted from original studies or an individualized data meta-analysis (43) using the method proposed by Parmar et al. (44). Study endpoints included OS, distant metastasis-free survival (DMFS) and locoregional recurrence-free survival (LRFS). Given the different definition of progression-free survival (PFS) in the trials, we did not included it into analysis.

### Statistical Method

First, we conducted pairwise meta-analysis comparison between each treatment group was using Stata 12.0 (StataCorp LP, College Station, TX, USA). Treatment effects were presented by HRs and corresponding 95% CIs. Study heterogeneity was determined using the *I*^2^ statistic or χ^2^ test. An *I*^2^ statistic > 50% or the *P*-value of χ^2^ test < 0.1 indicated statistically heterogeneity; otherwise, no heterogeneity exist between studies. Then, we performed network comparisons using the R (version 3.3.3; R Foundation, Vienna, Austria) *netmeta* package (45, 46). The frequentist approach (45) was adopted to carry out the network meta-analysis. Before multiple comparison, heterogeneity or inconsistency between treatment arms was assessed by Q test (45). If no significant heterogeneity existed (*P* > 0.1), fixed-effects model would be employed; otherwise, the random-effects model would be used. Finally, each treatment arm was ranked based on their corresponding P-score (47). A P-score of 100% suggested that treatment is the best, and a P-score of 0% indicated the worst treatment. Toxicity between different arms were compared using the χ^2^ test. A two-sided *P* < 0.05 was considered significant. Detailed process of multiple network comparison was shown in Supplementary Method.

## Conclusion

In summary, this network meta-analysis demonstrates IC+CCRT is superior to CCRT and provides highest benefit on OS, DMFS, and LRFS benefits LRFS. Therefore, IC+CCRT may be a better choice for advanced NPC at clinical practice. Head-to-head clinical trials comparing IC+CCRT with CCRT+AC are warranted to validate our findings.

## Author Contributions

ML and WY conceived and designed the experiments and conducted literature searching. ML, WY, J-DM, X-BZ, and D-KC extracted study data and performed analysis. ML, WY, LX, and L-FX contributed to reagents, materials, and analysis tools. ML, WY, and Y-BS wrote the paper, WY and YG contributed to quality control and review of the manuscript. All authors approved the final version of this manuscript.

### Conflict of Interest Statement

The authors declare that the research was conducted in the absence of any commercial or financial relationships that could be construed as a potential conflict of interest.
