# Supplementary material for: The Changing Role of Chemotherapy in Locoregionally Advanced Nasopharyngeal Carcinoma: A Updated Systemic Review and Network Meta-Analysis
Source: Front Oncol. 2018 Dec 19;8:597. doi: 10.3389/fonc.2018.00597 (PMC6305856; doi:10.3389/fonc.2018.00597)

**SUPPLEMENTARY METHODS**

**Study inclusion criteria**

1. Patients with newly, pathologically diagnosed locoregionally advanced NPC (WHO type 1 to 3) without previous chemotherapy or radiotherapy;
2. Randomized controlled phase II/III trials;
3. Patients received conventional-fractionation radiotherapy;
4. Radical radiation dosage should be delivered (with a total radiation dose of 66 Gy or more at conventional fraction);
5. Treatment arms should be IC+CCRT, CCRT+AC, CCRT or RT alone.
6. Concurrent chemotherapy should be platinum-based regimens.

**Literature search strategy**

The following electronic databases were searched to identify all eligible trials for this network meta-analysis: PubMed, Web of Science, Cochrane Library as well as Chinese literature databased of WanFang database and National Knowledge Infrastructure. Furthermore, it was supplemented by manual search of reference lists of all available primary studies, review articles, meeting reports and relevant books. Searching headlines included nasopharyngeal carcinoma or cancer or neoplasm, induction or neoadjuvant chemotherapy, concurrent chemoradiotherapy, adjuvant chemotherapy, and radiotherapy. The search was limited to randomized controlled trials but not English language.

**Quality control**

To assess the quality of included studies, we examined the randomization procedure, estimation of sample size, adoption of blind in study design, allocation concealment, if the intention-to-treat analysis being followed, loss to follow-up and dropout. Jadad/Oxford quality scoring system was adopted to quantify study quality [[1](#_ENREF_1)]. Any discrepancies would be solved by consensus.

**Data extraction**

Three investigators (M.L, W.Y and J-DM) reviewed the included studies and extracted the data independently. Data on study design, study time, number of patients in each arm, staging information, randomization scheme, follow-up duration, treatment protocol, endpoints and failure patterns were abstracted. Any discrepancies in quality assessment and data extraction were solved by consensus.

**Statistical analysis**

The primary endpoint of this network meta-analysis was OS, defined as the time from random assignment to death. Secondary endpoints were distant metastasis-free survival (DMFS) and locoregional recurrence-free survival (LRFS), defined as the time from random assignment to the first remote failure or locoregional, respectively. Results regarding the survival endpoints were expressed as hazard ratios (HRs), which was the only summary statistic allowed for both censoring and time to an event. If HR and its variance were available in an individual trial, then these data were directly used. If not, extraction of summary statistics was performed from two previous meta-analysis based on individualized data [[2](#_ENREF_2), [3](#_ENREF_3)] by using the methods detailed by Parmar et al. [[4](#_ENREF_4)].

Traditional pairwise meta-analyses were performed by using Stata statistical package 13.0 (StataCorp LP, College Station, TX, USA). We calculated the pooled estimates of HRs and 95% confidence intervals (CIs) of direct comparisons between two therapeutic regimens. A two-sided *P*-value of < 0.05 was considered significant. Heterogeneity across studies was tested by χ^2^ test and *I*^2^ statistic along with a forest plot. Statistically significant heterogeneity was defined as a χ^2^ *P*-value < 0.1 or an *I*^2^ statistic > 50%.

The network meta-analysis was planned in the R software (version 3.3.3; R Foundation, Vienna, Austria) using the *netmeta* package [[5](#_ENREF_5), [6](#_ENREF_6)] and frequentist approach [[5](#_ENREF_5)]. Logarithmic of HR (logHR) and its variance (selogHR) would be prepared for statistical data analysis of network meta-analysis. Treatment effects were estimated by HRs with corresponding 95% confidence intervals (CIs). Heterogeneity or inconsistency between and within designs was established by Q test which was proposed by Rücker et al. [[5](#_ENREF_5)] to be a generalization of Cochran’s test. No heterogeneity existed if *P* < 0.1, and fixed-effects model would be used. In case of significant heterogeneity, the use of random-effects model and the performance of sensitivity analysis would be considered. Forest plots of network meta-analysis were obtained using CCRT, CCRT + AC and IC + CCRT as the reference group, respectively. A P-score, proposed by Rücker and Schwarzer [[7](#_ENREF_7)] as a frequentist analog to surface under the cumulative ranking curve [[8](#_ENREF_8), [9](#_ENREF_9)] would be adopted to rank the treatment arms. P-score would be 100% for the best treatment and 0% for the worst treatment. Additionally, grade 3 or 4 toxicities were compared between different treatment arms using the χ^2^ test. A two-sided *P*-value of < 0.05 was considered significant.

**REFERENCE**

1. Jadad, A.R., Moore, R.A. & Carroll, D. Assessing the quality of reports of randomized clinical trials: is blinding necessary? *Control Clin Trials*. **17**, p. 1-12 (1996).

2. Blanchard, P., Lee, A. & Marguet, S. Chemotherapy and radiotherapy in nasopharyngeal carcinoma: an update of the MAC-NPC meta-analysis. *Lancet Oncol*. **16**, p. 645-55 (2015).

3. Ribassin-Majed, L., Marguet, S. & Lee, A.W. What Is the Best Treatment of Locally Advanced Nasopharyngeal Carcinoma? An Individual Patient Data Network Meta-Analysis. *J Clin Oncol*. JCO2016674119 (2016).

4. Parmar, M.K., Torri, V. & Stewart, L. Extracting summary statistics to perform meta-analyses of the published literature for survival endpoints. *Stat Med*. **17**, p. 2815-34 (1998).

5. Rucker, G. Network meta-analysis, electrical networks and graph theory. *Res Synth Methods*. **3**, p. 312-24 (2012).

6. Rucker, G., Schwarzer, G. & Krahn, U. Netmeta: Network meta-analysis using frequentist methods. <http://cran.r-project.org/web/packages/netmeta/index.html>

7. Rucker, G. & Schwarzer, G. Ranking treatments in frequentist network meta-analysis works without resampling methods. *BMC Med Res Methodol*. **15**, p. 58 (2015).

8. Chaimani, A., Higgins, J.P. & Mavridis, D. Graphical tools for network meta-analysis in STATA. *PLoS One*. **8**: e76654 (2013).

9. Salanti, G., Ades, A.E. & Ioannidis, J.P. Graphical methods and numerical summaries for presenting results from multiple-treatment meta-analysis: an overview and tutorial. *J Clin Epidemiol*. **64**, p. 163-71 (2011).

**SUPPLEMENTARY RESULTS**

The study by Kwong et al. is a factorial study which evaluated the value of concurrent and adjuvant chemotherapy in LA-NPC and contained four treatment arms: RT (Group A), CCRT (Group B), RT + AC (Group C) and CCRT + AC (group D). According to the inclusion criteria of our study, we took Group A, B and D into our network meta-analysis (Group A vs. B, Group A vs. D, Group D vs. B). Summary of the baseline characteristic of this study was presented in ***Supplementary Table S5***. After including this study, a total of 13 studies^1-13^, and 3416 patients were analyzed, with 1095 patients receiving CCRT, 897 patients receiving CCRT + AC, 555 patients receiving IC + CCRT and 869 patients receiving RT alone (***Supplementary Figure S1***). HRs and corresponding 95% CI were extracted from two previous meta-analysis based on individualized patient data^14-15^ and OS was available for all three comparisons, while DMFS and LRFS were available for only two comparisons (Group D vs. B and Group B vs. A).

***Supplementary Figure S2*** *presented the* results of pairwise meta-analysis. Intriguingly, there was no heterogeneity in all the comparisons and endpoints. Consistent with the results of previous pairwise meta-analysis, IC+CCRT achieved significantly better OS (HR, 0.63; 95% CI, 0.43-0.83), DMFS (HR, 0.57; 95% CI, 0.39-0.75) and LRFS (HR, 0.63; 95% CI, 0.36-0.89). Also, CCRT+AC was associated with significantly improved OS (HR, 0.63; 95% CI, 0.53-0.73), DMFS (HR, 0.51; 95% CI, 0.39-0.64) and LRFS (HR, 0.48; 95% CI, 0.32-0.64), and CCRT could significantly improve OS (HR, 0.77; 95% CI, 0.61-0.93) and DMFS (HR, 0.56; 95% CI, 0.39-0.74). No significant difference was observed between CCRT+AC and CCRT treatment arms.

Multiple treatment comparisons was shown in ***Supplementary Figure S1***. Results of network meta-analysis with different reference were presented in ***Supplementary Table S6*** and ***Supplementary Figure S3***. Similar as previous results, there was no heterogeneity and inconsistency within and between studies for all the endpoints, and fixed-effects model was therefore used. Generally, the multiple network meta-analysis results were consistent with previous. Compared to CCRT, IC+CCRT achieved significantly better OS (HR, 0.68; 95% CI, 0.51-0.92), DMFS (HR, 0.58; 95% CI, 0.44-0.78) and LRFS (HR, 0.67; 95% CI, 0.47-0.98). However, no significant survival differences were found between CCRT-AC and CCRT, or CCRT-AC and IC-CCRT. Notably, RT alone always led to significantly poorer survival outcomes compared with the other three treatments except RT vs. CCRT for LRFS (HR, 1.26; 95% CI, 0.93-1.69). The corresponding P-scores of IC+CCRT, CCRT+AC, CCRT and RT treatment arms were 94.5%, 71.2%, 34.1%, and 0.2% for OS; 99.6%, 57.7%, 42.7% and < 0.1% for DMFS; 86.6%, 78.2%, 32.8% and 2.4% for LRFS, indicating IC+CCRT has the highest probability of being the best treatment in terms of OS, DMFS and LRFS.

After including the study by Kwong et al, the conclusion still remained valid and IC + CCRT was even found to be superior to CCRT + AC for DMFS (HR, 0.63; 95% CI, 0.43-0.93). Therefore, IC + CCRT achieved the highest on OS, DMFS and LFRS.

**REFERENCE**

1. Al-Sarraf, M., LeBlanc, M. & Giri, P.G. Chemoradiotherapy versus radiotherapy in patients with advanced nasopharyngeal cancer: phase III randomized Intergroup study 0099. *J Clin Oncol*. **16**, p. 1310-7 (1998).
2. Cao, S.M., Yang, Q. & Guo, L. Neoadjuvant chemotherapy followed by concurrent chemoradiotherapy versus concurrent chemoradiotherapy alone in locoregionally advanced nasopharyngeal carcinoma: A phase III multicentre randomised controlled trial. *Eur J Cancer*. **75**, p. 14-23 (2017).
3. Chan, A.T., Leung, S.F. & Ngan, R.K. Overall survival after concurrent cisplatin-radiotherapy compared with radiotherapy alone in locoregionally advanced nasopharyngeal carcinoma. *J Natl Cancer Inst*. **97**, p. 536-9 (2005).
4. Chen, L., Hu, C.S. & Chen, X.Z. Adjuvant chemotherapy in patients with locoregionally advanced nasopharyngeal carcinoma: Long-term results of a phase 3 multicentre randomised controlled trial. *Eur J Cancer*. **75**, p. 150-8 (2017).
5. Chen, Y., Sun, Y. & Liang, S.B. Progress report of a randomized trial comparing long-term survival and late toxicity of concurrent chemoradiotherapy with adjuvant chemotherapy versus radiotherapy alone in patients with stage III to IVB nasopharyngeal carcinoma from endemic regions of China. *Cancer*. **119**, p. 2230-8 (2013).
6. Hui, E.P., Ma, B.B. & Leung, S.F. Randomized phase II trial of concurrent cisplatin-radiotherapy with or without neoadjuvant docetaxel and cisplatin in advanced nasopharyngeal carcinoma. *J Clin Oncol*. **27**, p. 242-9 (2009).
7. Kwong, D.L., Sham, J.S. & Au, G.K. Concurrent and adjuvant chemotherapy for nasopharyngeal carcinoma: a factorial study. *J Clin Oncol*. **22**, p. 2643-53 (2004).
8. Lee, A.W., Tung, S.Y. & Chan, A.T. A randomized trial on addition of concurrent-adjuvant chemotherapy and/or accelerated fractionation for locally-advanced nasopharyngeal carcinoma. *Radiother Oncol*. **98**, p. 15-22 (2011).
9. Lee, A.W., Tung, S.Y. & Chua, D.T. Randomized trial of radiotherapy plus concurrent-adjuvant chemotherapy vs radiotherapy alone for regionally advanced nasopharyngeal carcinoma. *J Natl Cancer Inst*. **102**, p. 1188-98 (2010).
10. Sun, Y., Li, W.F. & Chen, N.Y. Induction chemotherapy plus concurrent chemoradiotherapy versus concurrent chemoradiotherapy alone in locoregionally advanced nasopharyngeal carcinoma: a phase 3, multicentre, randomised controlled trial. *Lancet Oncol*. **17**, p. 1509-20 (2016).
11. Tan, T., Lim, W.T. & Fong, K.W. Concurrent chemo-radiation with or without induction gemcitabine, Carboplatin, and Paclitaxel: a randomized, phase 2/3 trial in locally advanced nasopharyngeal carcinoma. *Int J Radiat Oncol Biol Phys*. **91**, p. 952-60 (2015).
12. Wee, J., Tan, E.H. & Tai, B.C. Randomized trial of radiotherapy versus concurrent chemoradiotherapy followed by adjuvant chemotherapy in patients with American Joint Committee on Cancer/International Union against cancer stage III and IV nasopharyngeal cancer of the endemic variety. *J Clin Oncol*. **23**, p. 6730-8 (2005).
13. Wu, X., Huang, P.Y. & Peng, P.J. Long-term follow-up of a phase III study comparing radiotherapy with or without weekly oxaliplatin for locoregionally advanced nasopharyngeal carcinoma. *Ann Oncol*. **24**, p. 2131-6 (2013).
14. Blanchard, P., Lee, A. & Marguet, S. Chemotherapy and radiotherapy in nasopharyngeal carcinoma: an update of the MAC-NPC meta-analysis. *Lancet Oncol*. **16**, p. 645-55 (2015).
15. Ribassin-Majed, L., Marguet, S. & Lee, A.W. What Is the Best Treatment of Locally Advanced Nasopharyngeal Carcinoma? An Individual Patient Data Network Meta-Analysis. *J Clin Oncol*. JCO2016674119 (2016).

**SUPPLEMENTARY TABLES**

**Supplementary Table S1.** Quality assessment of the included 12 studies using revised Jadad scale.

| Study | Randomization process | Allocation concealment | Blinding | Dropout | Jadad score |
| --- | --- | --- | --- | --- | --- |
| **IC + CCRT vs. CCRT** |  |  |  |  |  |
| Hui et al. 2009 | Yes | Yes | No | Yes | 5 |
| Frikha et al. 2017 | Yes | Yes | No | Yes | 4 |
| Sun et al. 2016 | Yes | Yes | No | Yes | 5 |
| Cao et al. 2017 | Yes | Yes | No | Yes | 5 |
| **CCRT + AC vs. RT** |  |  |  |  |  |
| Al-Sarraf et al. 1998 | No | No | No | Yes | 1 |
| Wee et al. 2005 | Yes | Yes | No | Yes | 4 |
| Lee et al. 2005,2010 | Yes | Yes | No | Yes | 4 |
| Lee et al. 2006,2011 | Yes | Yes | No | Yes | 4 |
| Chen et al. 2008,2013 | Yes | Yes | Yes | Yes | 6 |
| **CCRT + AC vs. CCRT** |  |  |  |  |  |
| Chen et al. 2012,2017 | Yes | Yes | No | Yes | 5 |
| **CCRT vs. RT** |  |  |  |  |  |
| Chan et al. 2002,2005 | Yes | Yes | No | Yes | 5 |
| Zhang et al. 2005,2013 | No | No | No | Yes | 1 |

Abbreviations: CCRT = concurrent chemoradiotherapy; AC = adjuvant chemotherapy; RT = radiotherapy; IC = induction chemotherapy.

**Supplementary Table S2**. League table presenting the results of direct meta-analysis (upper right triangle) and network meta-analysis (lower left triangle) for overall survival using fixed-effects model.

| CCRT (1) | 0.83  (0.50-1.15) | 0.63  (0.43-0.83) | 0.75  (0.58-0.91) |
| --- | --- | --- | --- |
| 0.86  (0.69-1.07) | CCRT + AC (2) | - | 0.63  (0.53-0.74) |
| 0.69  (0.51-0.92) | 0.80  (0.55-1.15) | IC + CCRT (3) | - |
| 1.31  (1.08-1.59) | 1.52  (1.31-1.77) | 1.91  (1.34-2.70) | RT (4) |

Abbreviations: CCRT = concurrent chemoradiotherapy; AC = adjuvant chemotherapy; IC = induction chemotherapy; RT = radiotherapy.

The cells contain hazard ratio (HR) and its 95% confidence interval of treatment comparison. For direct meta-analysis, the comparison should be CCRT + AC vs CCRT, IC + CCRT vs. CCRT, CCRT vs. RT and CCRT + AC vs. RT. With regard to network meta-analysis, the treatment groups with higher-number were compared to treatment groups with lower number.

**Supplementary Table S3**. League table presenting results of direct meta-analysis (upper right triangle) and network meta-analysis (lower left triangle) for distant metastasis-free survival using fixed-effects model.

| CCRT (1) | 0.81  (0.46-1.16) | 0.57  (0.39-0.75) | 0.61  (0.42-0.81) |
| --- | --- | --- | --- |
| 0.85  (0.65-1.12) | CCRT + AC (2) | - | 0.51  (0.39-0.64) |
| 0.58  (0.44-0.78) | 0.68  (0.46-1.02) | IC + CCRT (3) | - |
| 1.47  (1.14-1.89) | 1.72  (1.40-2.12) | 2.52  (1.72-3.70) | RT (4) |

Abbreviations: CCRT = concurrent chemoradiotherapy; AC = adjuvant chemotherapy; IC = induction chemotherapy; RT = radiotherapy.

The cells contain hazard ratio (HR) and its 95% confidence interval of treatment comparison. For direct meta-analysis, the comparison should be CCRT + AC vs. CCRT, IC + CCRT vs. CCRT, CCRT vs. RT and CCRT + AC vs. RT. With regard to network meta-analysis, the treatment groups with higher-number were compared to treatment groups with lower number.

**Supplementary Table S4**. League table presenting results of direct meta-analysis (upper right triangle) and network meta-analysis (lower left triangle) for locoregional recurrence-free survival using fixed-effects model.

| CCRT (1) | 0.91  (0.36-1.46) | 0.63  (0.36-0.89) | 0.86  (0.49-1.23) |
| --- | --- | --- | --- |
| 0.74  (0.51-1.08) | CCRT + AC (2) | - | 0.48  (0.32-0.64) |
| 0.67  (0.47-0.98) | 0.91  (0.54-1.54) | IC + CCRT (3) | - |
| 1.25  (0.89-1.76) | 1.68  (1.28-2.21) | 1.85  (1.12-3.06) | RT (4) |

Abbreviations: CCRT = concurrent chemoradiotherapy; AC = adjuvant chemotherapy; IC = induction chemotherapy; RT = radiotherapy.

The cells contain hazard ratio (HR) and its 95% confidence interval of treatment comparison. For direct meta-analysis, the comparison should be CCRT + AC vs. CCRT, IC + CCRT vs. CCRT, CCRT vs. RT and CCRT + AC vs. RT. With regard to network meta-analysis, the treatment groups with higher-number were compared to treatment groups with lower number.

**Supplementary Table S5**. Baseline characteristics of the study by Kwong et al.

| Study | No. of patients | Study time | Median follow-up duration (months) | Patient stage | Radiotherapy |  | Chemotherapy |  |
| --- | --- | --- | --- | --- | --- | --- | --- | --- |
|  |  |  |  |  |  | Induction | Concurrent | Adjuvant |
| RT arm (Group A) | 55 | 1995-2001 | 37 | AJCC II-IV, any T, any N, M0 | 66-68 Gy at 2.0 or 2.5 Gy/f/daily (4 or 5f/qw) + 10 Gy boost dose to parapharyngeal extension or residual neck node | None | None | None |
| CCRT (Group B) | 56 | 1995-2001 | 37 | AJCC II-IV, any T, any N, M0 | 66-68 Gy at 2.0 or 2.5 Gy/f/daily (4 or 5f/qw) + 10 Gy boost dose to parapharyngeal extension or residual neck node | None | UFT 200mg TID, 7 days per week for 5 to 8 weeks | None |
| RT + AC (Group C) | 54 | 1995-2001 | 37 | AJCC II-IV, any T, any N, M0 | 66-68 Gy at 2.0 or 2.5 Gy/f/daily (4 or 5f/qw) + 10 Gy boost dose to parapharyngeal extension or residual neck node | None | None | Cisplatin 100 mg/m^2^ d1 + 5-FU 1000mg/m^2^/d d1-d3 civ, q3w × 6  or Vincristine 2 mg d1 + Bleomycin 30 mg d1 + Methotrexate 150 mg/m^2^ d1, q3w × 6 |
| CCRT + AC (Group D) | 57 | 1995-2001 | 37 | AJCC II-IV, any T, any N, M0 | 66-68 Gy at 2.0 or 2.5 Gy/f/daily (4 or 5f/qw) + 10 Gy boost dose to parapharyngeal extension or residual neck node | None | UFT 200mg TID, 7 days per week for 5 to 8 weeks | Cisplatin 100 mg/m^2^ d1 + 5-FU 1000mg/m^2^/d d1-d3 civ, q3w × 6  or Vincristine 2 mg d1 + Bleomycin 30 mg d1 + Methotrexate 150 mg/m^2^ d1, q3w × 6 |

Abbreviations: CCRT = concurrent chemoradiotherapy; AC = adjuvant chemotherapy; RT = radiotherapy; AJCC = American Joint Committee on cancer; f = fraction; 5-FU = 5-fluorouracil; civ = continuous i.v.; q3w = every 3 weeks.

**Supplementary Table S6**. Summary of network meta-analysis results after including the study by Kwong et al.

| Treatment arm | OS | DMFS | LRFS |
| --- | --- | --- | --- |
| *P* value of Overall heterogeneity/inconsistency | 0.65 | 0.30 | 0.69 |
| *P* value of heterogeneity (within designs) | 0.58 | 0.23 | 0.63 |
| *P* value of heterogeneity (between designs) | 0.68 | 0.93 | 0.61 |
| CCRT |  |  |  |
| HR | 1.00 | 1.00 | 1.00 |
| P-score (%) | 34.1 | 42.7 | 32.8 |
| CCRT+AC |  |  |  |
| HR (95% CI) | 0.82 (0.67-1.00) | 0.92 (0.71-1.20) | 0.73 (0.52-1.02) |
| P-score (%) | 71.2 | 57.7 | 78.2 |
| IC+CCRT |  |  |  |
| HR (95% CI) | 0.68 (0.51-0.92) | 0.58 (0.44-0.78) | 0.67 (0.47-0.98) |
| P-score (%) | 94.5 | 99.6 | 86.6 |
| RT |  |  |  |
| HR (95% CI) | 1.25 (1.05-1.49) | 1.58 (1.24-2.00) | 1.26 (0.93-1.69) |
| P-score (%) | 0.2 | < 0.01 | 2.4 |

Abbreviations: OS = overall survival; DMFS = distant metastasis-free survival; LRFS = locoregional recurrence-free survival; CCRT = concurrent chemoradiotherapy; AC = adjuvant chemotherapy; IC = induction chemotherapy; RT = radiotherapy. HR = hazard ratio; CI = confidence interval.

Fixed-effects model was used for overall survival, distant metastasis-free survival and locoregional recurrence-free survival.

**SUPPLEMENTARY FIGURES**

**Supplementary Figure S1**. Graphical presentation of the trial network for overall survival. The width of the lines between nodes is proportional to the number of comparisons. Only two treatment arms receiving conventional-fraction radiotherapy in the study by Lee et al. (2011) were included in this study. CCRT, concurrent chemoradiotherapy; IC, induction chemotherapy; AC, adjuvant chemotherapy; RT, radiotherapy.


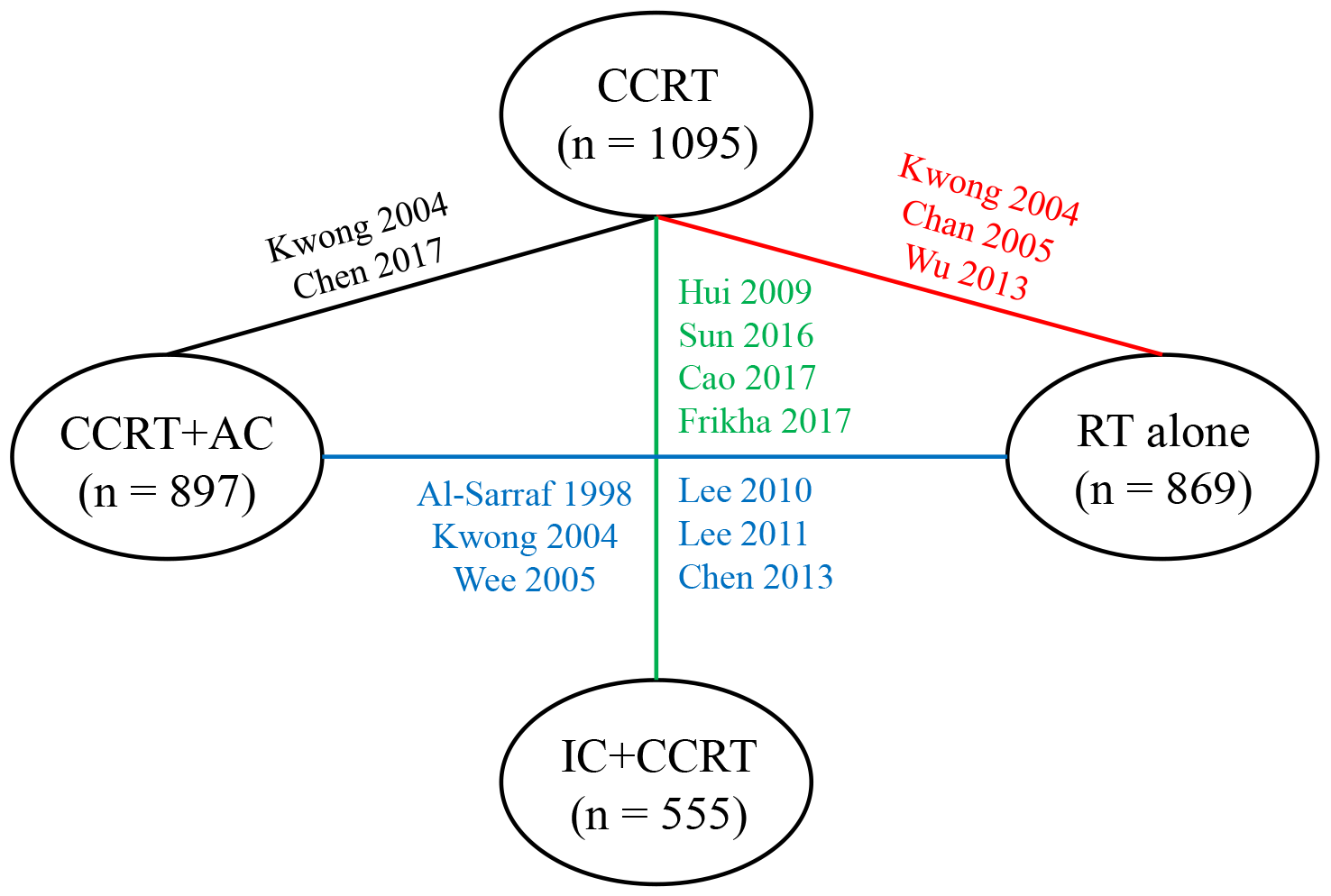


**Supplementary Figure S2**. Results of pairwise meta-analysis. CCRT, concurrent chemoradiotherapy; IC, induction chemotherapy; AC, adjuvant chemotherapy; RT, radiotherapy; HR, hazard ratio; CI, confidence interval.


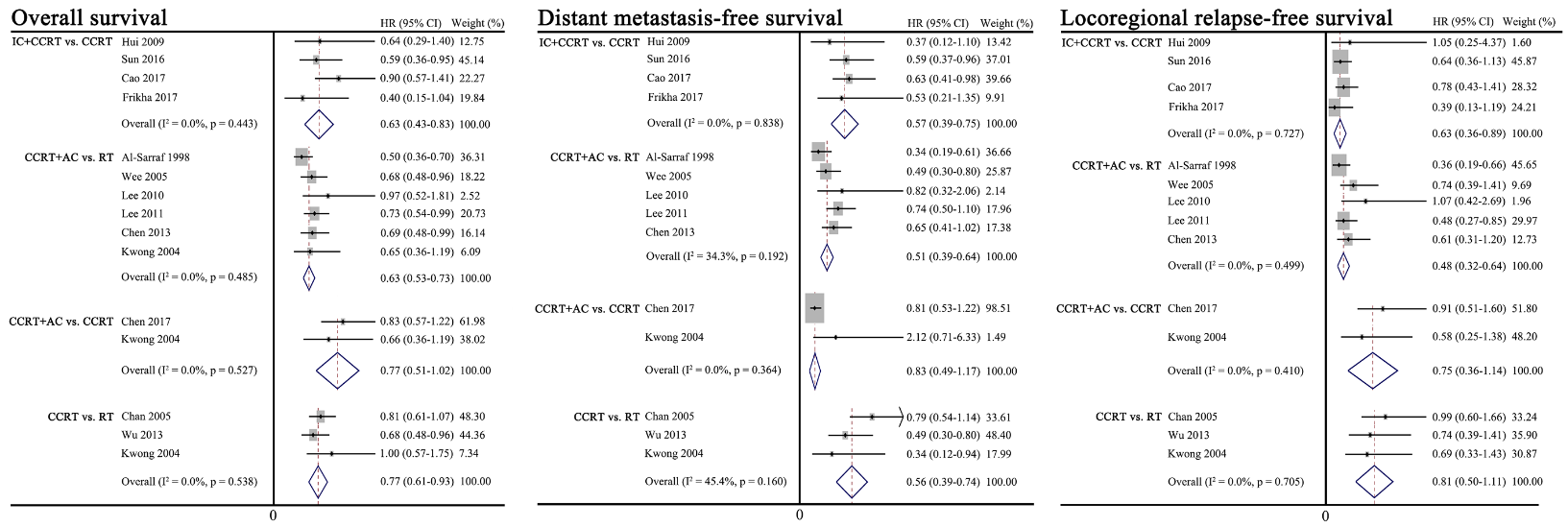


**Supplementary Figure S3**. Forest plot of network meta-analysis for overall survival, distant metastasis-free survival and locoregional recurrence-free survival with different reference groups. CCRT, concurrent chemoradiotherapy; IC, induction chemotherapy; AC, adjuvant chemotherapy; RT, radiotherapy.


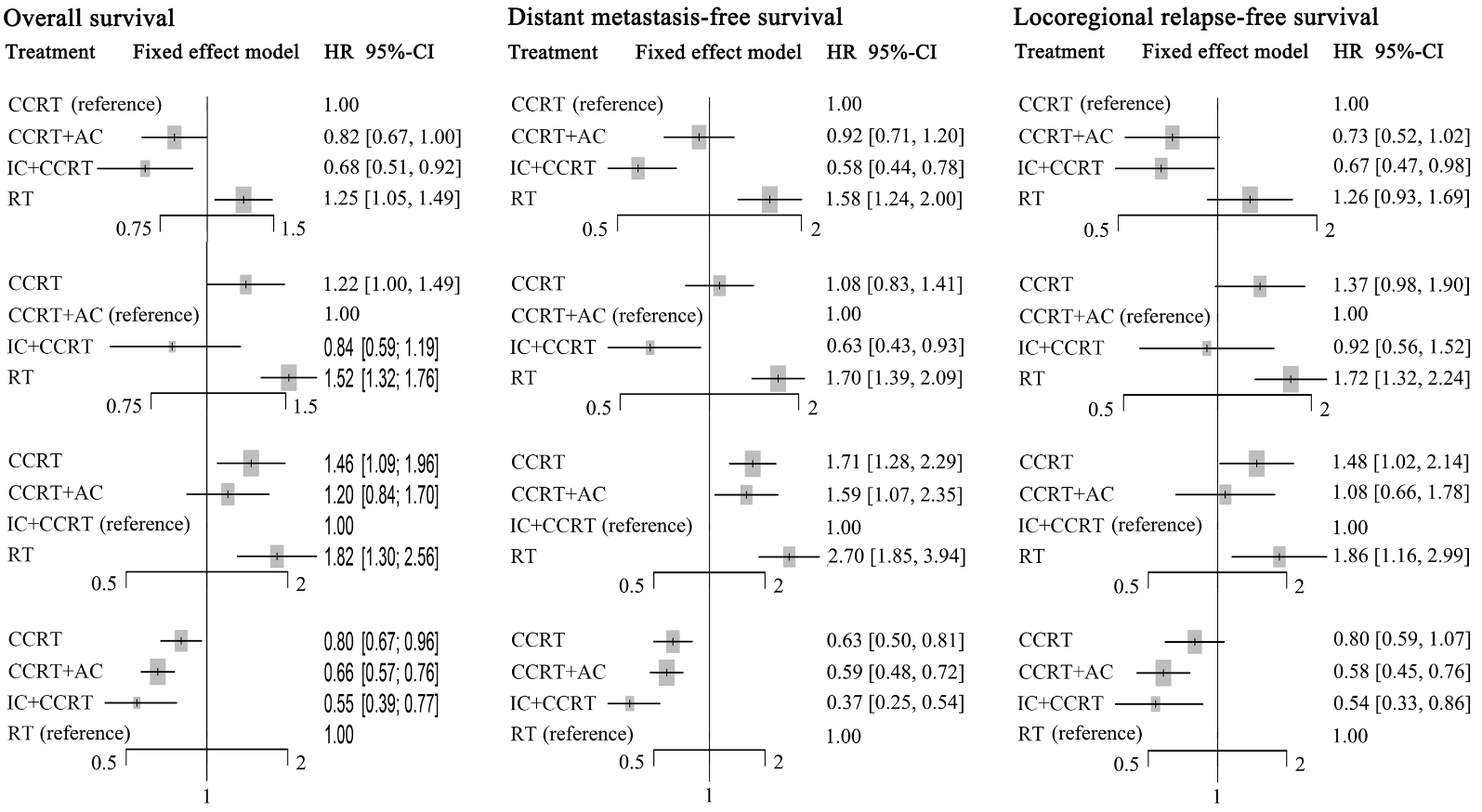

Supplement: Supplementary file 1 [file Data_Sheet_1.docx]
